# Transcatheter Tricuspid Interventions: Past, Present, and Future

**DOI:** 10.14797/mdcvj.1250

**Published:** 2023-05-16

**Authors:** Colin M. Barker, Kashish Goel

**Affiliations:** 1 Vanderbilt Heart and Vascular Institute, Vanderbilt University Medical Center, Nashville, Tennessee, US

**Keywords:** tricuspid regurgitation, tricuspid valve: transcatheter tricuspid valve repair, TTVR

## Abstract

Tricuspid regurgitation (TR) etiologies include primary valve pathology or secondary (functional) regurgitation from increased hemodynamic pressure or volume on the right side of the heart. Patients with severe TR have a worse prognosis independent of all other variables. Surgical treatment for TR has mostly been limited to patients undergoing concomitant left-sided cardiac surgery. The results and durability of surgical repair or replacement are not well defined. For patients with significant and symptomatic TR, transcatheter techniques would be beneficial, but these techniques and devices have been slow to develop. Much of the delay is a result of neglect and challenges in defining the symptoms associated with TR. In addition, the anatomic and physiological aspects of the tricuspid valve apparatus present unique challenges. Several devices and techniques are in various phases of clinical investigation. This review highlights the current landscape of transcatheter tricuspid interventions and future opportunities. It is imminent that these therapies become commercially available and widely adopted to have a significant positive impact on millions of patients that have been neglected.

## Introduction

Tricuspid regurgitation (TR) is a condition in which the tricuspid valve, located on the right side of the heart, fails to close properly. This leads to the backflow of blood from the right ventricle of the heart into the right atrium during systole. The mechanism can be complex, multifactorial, and anatomically variable. The impact of isolated TR has been challenging to quantify. Analysis of contemporary big data shows that the effect of TR is marginal regarding mortality but has a significant negative effect on quality of life and is a tremendous healthcare burden in terms of cost and utilization of resources.^1^,^2^,^3^,^4^

There are three types of TR, each with different etiologies: primary, secondary, and cardiac electric implantable device-related (CEID).^5^ Primary TR is congenital or acquired and occurs when there is a problem with the tricuspid valve itself, such as Ebstein’s anomaly, damage from infection, carcinoid, or trauma. Secondary TR is due to conditions that increase the volume or pressure on the right side of the heart or change the morphology of the right-sided chambers^6^; this includes pulmonary hypertension, left-sided heart failure, or right atrial enlargement with annular dilation. Finally, CEID-related TR is a result of right ventricle pacing or direct interaction between the device and the tricuspid apparatus interfering with normal valve function.

When the tricuspid valve fails to close properly, blood flows back into the right atrium, causing volume overload on the right side of the heart. This causes enlargement of the right atrium and right ventricle, leading to reduced cardiac output and right-sided heart failure. Furthermore, retrograde flow can lead to symptoms such as shortness of breath, fatigue, swelling in the legs and abdomen, and ultimately liver and renal failure from congestion.

Tricuspid regurgitation can be diagnosed through physical examination, echocardiography, or other imaging tests. Treatment depends on the severity of the lesion and may include diuretics to control symptoms, surgery to repair or replace the tricuspid valve, or treatment of the underlying condition causing the regurgitation.

Given the anatomical challenges of the tricuspid valve and subvalvular apparatus, isolated tricuspid valve surgery has been an unattractive option for both patients and providers. Furthermore, given lack of any impact on mortality for isolated tricuspid valve disease compared with the high-mortality risk of cardiac surgery, this option is hardly ever justified. As a result, isolated tricuspid valve surgery has been avoided in most circumstances. With the rapid increase in innovation, effectiveness, and superior outcomes of transcatheter interventions for heart valve disease, tricuspid valve interventions have become a hot topic with a promising potential impact on patient care and the healthcare system.^7^,^8^,^9^

## Tricuspid Apparatus Anatomy

The tricuspid valve is part of the tricuspid apparatus, which is responsible for regulating the flow of blood from the systemic venous return through the right atrium into the right ventricle of the heart. The tricuspid apparatus is comprised of several structures:

1. Tricuspid valve: The tricuspid valve consists of multiple defined leaflets or cusps (anterior, posterior, and septal) that open and close to allow blood flow from the right atrium to the right ventricle during diastole and prevent backflow during systole. Anywhere from two to five independent leaflets can create the tricuspid valve.
2. Chordae tendineae: These fibrous cords of various lengths connect the free edges of the tricuspid valve cusps to the papillary muscles located in the walls of the right ventricle. The chordae tendineae help maintain the correct position and tension of the valve leaflets during the cardiac cycle. Histologically, tricuspid chordae are composed of collagen bundles that are less extensible compared with mitral valve chordae of similar sizes.
3. Papillary muscles: Three papillary muscles located in the right ventricle are attached to the chordae tendineae. These muscles contract during systole to prevent the valve leaflets from bulging back into the right atrium and causing regurgitation.
4. Annulus: The annulus is a D-shaped, non-planar structure that supports the tricuspid valve and provides a base for the leaflets to attach. The annulus lacks rigid fibrous tissue and instead has muscle bands, including epicardium and endocardium. This is a highly dynamic structure with a change in area of up to 30% during the cardiac cycle.

## Patient Evaluation

The assessment and evaluation of tricuspid valve function and associated conditions typically involve a combination of medical history, physical examination, and diagnostic tests. There are challenges in evaluating patients with TR and assessing its clinical impact. Specifically, defining symptoms, which can often be vague and systemic, and predicting a response to intervention can be difficult. The symptoms can be atypical compared to other cardiovascular disease processes. The typical chest pain, shortness of breath, and syncope are unusual in tricuspid regurgitation. Years of conventional teaching and neglect of TR should be challenged and reassessed since the detrimental impact of delaying TR treatment is becoming obvious.

Given the unique clinical manifestations of tricuspid regurgitation, more comprehensive assessments are warranted when a patient is referred for consultation and evaluation for intervention. Not all patients showing severe TR on an echocardiogram need an intervention. Likewise, a patient does not need pulsatile jugular veins or refractory ascites or edema to trigger an evaluation for intervention. In fact, these findings may indicate that it is too late for an intervention and the window of opportunity may have passed. Thus, routine assessment of frailty, quality of life, and functional capacity are mandatory during initial evaluation and consultation.

## Transcatheter Tricuspid Interventions

Despite overwhelming evidence for safety and effectiveness of multiple catheter-based strategies and devices to treat tricuspid valve disease, nothing is currently “approved” by the United States (US) Food and Drug Administration as a primary strategy for tricuspid valve replacement or repair (Table 1).^10^,^11^,^12^,^13^ The hypocrisy of the different pathways for surgical device replacement versus catheter-based interventions with identical devices remains and is enabled by society guidelines and recommendations.^14^ Whereas surgical device replacement requires no clinical evidence, catheter-based therapies require randomized controlled trials with several hundred patients, costing hundreds of millions of dollars. This double standard does not seem to be changing anytime soon, thereby continuing to delay patient access to effective therapies.^15^,^16^,^17^ Despite these challenges, several trials are actively underway. The first of these trials, TRILUMINATE, was recently presented and published.

**Table 1:** Transcatheter tricuspid valve interventions: past, present, and future.


REPAIR	**Coaptation Enhancement**	**Annuloplasty**

	Tri-Clip Pascal Dragonfly Mitralix	Trialign Cardioband TriCinch Millipede MIA PASTA Da Vingi

REPLACEMENT	**Orthotopic**	**Heterotopic**

	Evoque Intrepid V-Dyne Navigate Trisol Lux Topaz	TricValue TriCento Trillium

ALTERNATIVE	**Spacer**	

	Forma Croi Coramaze Tri-Flow	


### Transcatheter Tricuspid Valve Repair

**Figure 1:**
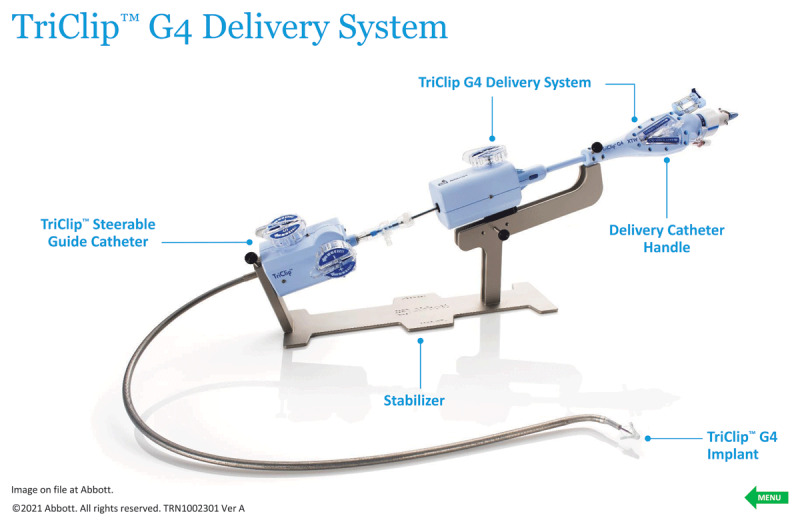
The Abbott TriClip G4 Delivery System and edge-to-edge repair device. Reproduced with permission of Abbott, © 2023. All rights reserved.

**Figure 2:**
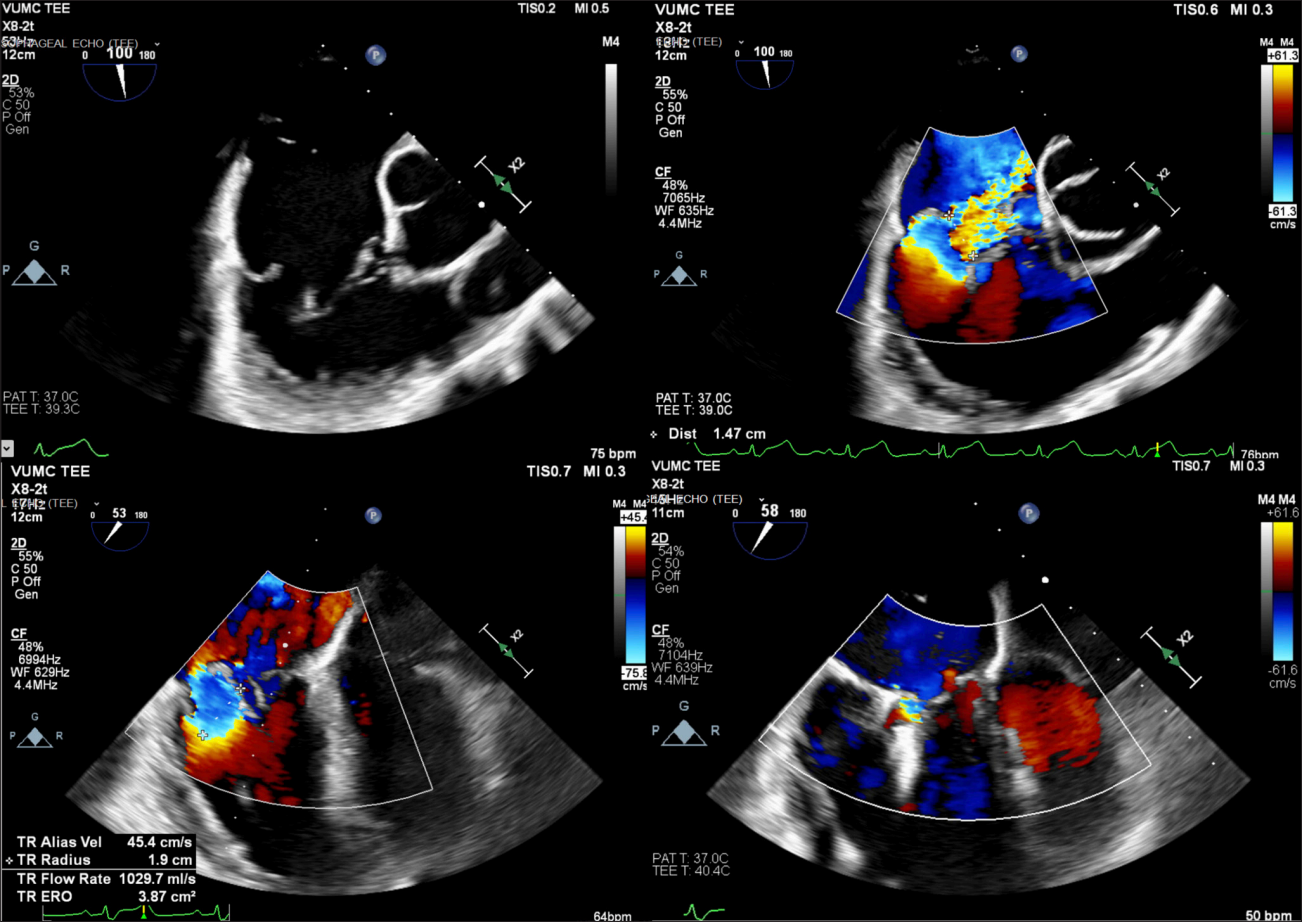
Transesophageal echocardiography intraprocedural image showing TriClip tricuspid valve repair. The tricuspid regurgitation went from torrential to trace after one anterior-posterior leaflet device and a second in the posterior-septal location.

The TRILUMINATE Pivotal Trial was designed to evaluate the safety and effectiveness of transcatheter tricuspid valve (TTV) repair with the TriClip™ device (Abbott Vascular, Figure 1) in symptomatic patients with severe TR who are at intermediate or greater estimated risk for mortality with tricuspid valve surgery.^18^,^19^ This was a multicenter, international, prospective, randomized controlled trial designed to test the superiority of TriClip therapy in addition to medical therapy (device group) over medical therapy alone (control group). The primary end point was a composite of mortality or tricuspid valve surgery, heart failure hospitalizations, and quality of life improvement ≥ 15 points assessed using the Kansas City Cardiomyopathy Questionnaire (KCCQ), evaluated at 12 months in a hierarchical fashion. In the analysis, the authors used a win ratio, which is a method for reporting composite end points and gives priority to the more clinically important events, such as mortality. The win ratio for the primary end point favored the intervention group versus the control (1.48; 95% CI, 1.06-2.13; *P* = .02). The components of the primary end point showed no significant differences in all-cause mortality, need for tricuspid valve surgery, or hospitalizations for heart failure between the device and control groups. The end point was driven entirely by change in KCCQ score between the two groups. The trial was not blinded or sham controlled. It is likely that those in the treatment arm felt like they “won” the randomization lottery as opposed to the controls, who may have been frustrated to have “lost.” These sentiments may have led to the difference in KCCQ that drove the entire trial, and there may be no benefit of this device in the population studied. This assessment is supported by the lack of difference in 6-minute walk test between groups. Regardless of the regulatory decisions moving forward based on the TRILUMINATE data, an objective appraisal of the results of this trial and intervention does not translate into an impactful opportunity to treat patients with TR. Conversely, there were no significant adverse events or increased rate of adverse events in the device group when compared to control, and thus the concept remains viable but still unproven (Figure 2).

The CLASP II TR trial is evaluating the safety and effectiveness of the PASCAL (Edwards Lifesciences) Transcatheter Repair System in patients with symptomatic severe TR who have been determined by a cardiac surgeon, with concurrence by the local heart team, to be at an intermediate or greater estimated risk of mortality with tricuspid valve surgery.^20^ The primary outcome is a composite end point including all-cause mortality, right ventricular assist device implantation or heart transplant, tricuspid valve intervention, heart failure hospitalizations, and quality of life improvement (measured by KCCQ score) at 2 years. The CLASP TR early feasibility study reported the 30-day data of the US single-arm, multicenter, prospective study of the PASCAL transcatheter valve repair system in the treatment of TR.^21^ Of the 34 patients enrolled in the study, 89% improved to NYHA functional class I/II (*P* < .001), the mean 6-minute walk distance improved by 71 m (*P* < .001), and the mean KCCQ score improved by 15 points (*P* < .001). The randomized trial, CLASP II TR, is currently enrolling and randomizing patients 1:1 to device versus medical therapy alone.

Transcatheter annuloplasty has been explored with various technologies for TR (Table 2), but none have been able to achieve the efficacy or durability needed to move forward in research and development. Several treatment opportunities exist regarding percutaneous annuloplasty, such as the prevalence of atrial functional TR and the potential to provide standalone therapy or combined strategies with other technologies (such as TTV repair) while also preserving future options for transcatheter interventions without disrupting the tricuspid leaflets. Despite the enthusiasm, many challenges and disappointments have confronted and stalled the progress of transcatheter annuloplasty repair—the most important being anchor/suture detachment, which remains common and is a fatal flaw. Furthermore, the proximity of the right coronary artery, the larger annular size of the tricuspid valve, challenging and complicated procedural imaging guidance, and ultimately the lack of reproducibility and adoptability have slowed the development of most annuloplasty therapies. In retrospect, this may not be surprising given the disappointing experience with surgical annuloplasty repair for TR.

**Table 2:** Benefits and risks of transcatheter tricuspid valve repair versus transcatheter tricuspid valve replacement. TR: tricuspid regurgitation; SLDA: single leaflet device attachment


REPAIR	REPLACEMENT

Less invasive, safer	More invasive

More residual TR	TR eliminated

Low to no pacemaker risk	High risk for pacemaker

Allows for future intervention	Limited options when fails

Risk of SLDA	Risk of mortality, bleeding

No need for anticoagulation	Needs anticoagulation

Limited by anatomy	Less dependent on anatomy


### Transcatheter Tricuspid Valve Replacement

TTV replacement (TTVR) is a minimally invasive procedure used to replace a malfunctioning tricuspid valve in patients who are not candidates for traditional open-heart surgery.^22^ TTVR is an evolving technology and procedure that is still being evaluated in clinical trials. Candidates for TTVR trials are typically patients who have tricuspid valve disease that is severe enough to cause symptoms but who are unable to undergo traditional open-heart surgery due to age, frailty, or other health conditions. Preprocedure evaluation is complex and requires familiarity and the ability to combine multimodality imaging techniques to predict procedural candidacy and device selection. TTVR is much less invasive and an attractive alternative to traditional open-heart surgery, but it is still a complex procedure that carries some risks. However, once refined, this intervention will have a role in managing all patients with TR. As opposed to the current transcatheter repair options, the TTVR devices have the advantage of consistently eliminating TR. What remains to be proven is if eliminating the TR in these patients translates to meaningful clinical improvement.

#### Orthotopic

The list of devices and procedural animations for orthotopic TTVR devices continues to expand and has surpassed the number of devices currently being evaluated for transcatheter mitral valve replacement (Table 2). To be successful, durable, and widely adopted, these devices will need to be administered percutaneously through the femoral vein. Given the lack of anatomical landmarks as outlined above, different strategies to incorporate different components of the tricuspid apparatus are used for deployment and stabilization. In addition, intraprocedural imaging is much more challenging and complicated compared with other structural or percutaneous valve interventions. Having an interventional imager with the skill set to guide and dictate the cadence of these procedures is essential for this procedure.

The NaviGate transcatheter valve (NaviGate Cardiac Structures Inc.) is a nitinol self-expanding tapered stent with a trileaflet equine pericardial valve. The ventricular side is anchored with 12 tynes to grasp native leaflets, with 12 atrial winglets on the atrial side. The current generation requires access with a 42F system either through the internal jugular vein or directly into the right atrium.^23^ In a report of 32 patients treated with the NaviGATE system, implantation success was 100%, with all experiencing a ≥ 2 grade reduction in TR severity grade and a 30-day mortality of 12.5%.^24^

The EVOQUE tricuspid valve replacement system (Edwards Lifesciences) is comprised of bovine pericardial leaflets with an intra-annular sealing skirt and anchors. The EVOQUE system is on a 28F delivery catheter and delivered via transfemoral venous access; the multiplanar steerable delivery system allows for coaxial deployment of the valve. The feasibility and safety of the EVOQUE tricuspid valve replacement system and its impact on short-term clinical outcomes was recently published, with 92% technical success and no intraprocedural mortality or conversion to surgery.^25^ At 30-day follow-up, mortality was 0%, 76% of patients were in NYHA functional class I or II, and TR grade was ≤ 2+ in 96% of patients. Major bleeding occurred in three patients (12%), two patients (8%) required pacemaker implantation, and one (4%) required dialysis. The TRISCEND II pivotal trial, which randomized patients with severe TR to EVOQUE versus medical therapy alone, was recently completed. It has a 2-year follow-up period and will provide clarity regarding the potential for this system to treat patients with symptomatic TR.

The Cardiovalve (Cardiovalve Ltd) is a TTVR system based on a low-profile frame made of three bovine pericardial leaflets and is delivered through a 32F transfemoral transvenous approach.^26^ The valve is composed of a dual self-expanding nitinol frame that creates 24 grasping points for native valve anchoring. The two distinct atrial and ventricular frames are welded together, and the atrial frame has a Dacron fabric-covered flange for improved sealing and anchoring. Currently, a handful of published case reports and presentations at scientific meetings describe early experience with the Cardiovalve for TR.^27^

The LuX-Valve (Ningbo Jenscare Biotechnology Co.) is a novel radial force–independent orthotopic TTVR device. The LuX-Valve consists of four components: (1) a trileaflet prosthetic valve with bovine pericardium; (2) a self-expandable nitinol valve stent consisting of an atrial disc; (3) one interventricular septal anchor “tongue”; and (4) two expanded polytetrafluoroethylene-covered graspers. The LuX-Valve is delivered via a 32F catheter through a right thoracotomy and transatrial approach under the guidance of transesophageal echocardiography and fluoroscopy. A prospective and observational first-in-man study reported the initial experience of 12 patients who received compassionate LuX-Valve implantation for the treatment of severe TR.^28^ Despite one mortality and significant perioperative morbidity (acute kidney injury and conversion to open surgical replacement), transthoracic echocardiography at 30 days showed none-to-mild residual TR in all but one patient (90.9%). Significant symptomatic improvement was observed with improved 6-minute walk tests (377 m versus 277 m) and NYHA functional status (54.5% at NYHA functional class II; *P* < .05). This is encouraging data in a sick cohort, but the procedure needs to be transfemoral to advance further in clinical settings.

#### Heterotopic

The concept of heterotopic valve implantation to treat regurgitant lesions has been around for 80 years. Charles Hufnagel designed a methacrylate chamber containing a methacrylate ball that was implanted in the descending aorta of a patient with aortic regurgitation in the 1940s and, subsequently, more than 200 patients were treated.^29^ Heterotopic heart valves to treat TR were adopted early in the transcatheter heart valve evolution. Specifically, Sapien valves designed to treat aortic stenosis were being deployed in the inferior vena cava to palliate refractory TR in those with no other options. This fell out of favor due to lack of effectiveness and durability as well as high mortality. In the interim, several innovations have evolved in this field. These devices are agnostic to the etiology of the TR and may have a palliative role when patients have no other option.

The TricValve system (OrbusNeich P&F) is a dedicated caval valve implantation device consisting of two self-expanding nitinol stents that harbor bovine pericardial leaflets, one specifically designed for the superior vena cava and another for the inferior vena cava.^30^ TRICUS EURO (Safety and Efficacy of the TricValve® Transcatheter Bicaval Valves System in the Superior and Inferior Vena Cava in Patients With Severe Tricuspid Regurgitation) was a CE mark trial testing the safety and efficacy of this system in patients with severe symptomatic TR deemed at high surgical risk.^31^,^32^ Thirty-five patients were treated using the TricValve system. At 30 days, procedural success was 94%, with no procedural deaths or conversions to surgery. A significant increase in quality of life at 6-month follow-up was observed, with improvement of KCCQ score from 42 at baseline to 59 at 6 months (*P* = .004), correlating with a significant improvement in NYHA functional class, with 79.4% of patients noted to be in functional class I or II at 6 months (*P* = .006). The rates of 6-month all-cause mortality and heart failure hospitalization were 8.5% and 20%, respectively. The US randomized controlled trial, called TRICAV, will soon be launched and will help define the potential role of this technology in treating TR.

Several other platforms in this class are in early development, such as CroíValve (CroíValve). The TricValve® (SingHealth Group), pending trial and data, will define the utility and potential role for this strategy in treating TR. Ultimately, the concept of heterotopic therapy for TR may have a role when TTV repair and TTVR systems have failed with recurrence of progressive symptomatic TR.^33^ It is unlikely that this will be a primary intervention for severe TR.

### Repair Versus Replacement

With the crowded spectrum of potential devices for replacement and repair for TR, decisions and guidance for which device to use will depend on individual patient characteristics and anatomy (Table 2). Ultimately, an algorithm that more clearly defines the optimal strategy will be determined by ongoing clinical trials, innovation, and experience. Currently, one strategy does appear to have some potential advantages over another. Specifically, repair may be more appealing in those with mobile leaflets, right ventricle dysfunction, and contraindication for anticoagulation in the setting of excellent imaging quality. Alternatively, replacement may be superior in the setting of a large leaflet gap with tethered cords and potentially in the setting of lead-induced tricuspid regurgitation.

Annuloplasty devices, spacers, and heterotopic valve replacement therapies will be limited and unlikely to be the primary intervention for transcatheter therapies in severe, symptomatic TR.^30^,^34^ These devices are limited by durability, the anatomy of right-sided structures (including proximity of important structures such as the right coronary artery, coronary sinus, and conduction system), variability in the caval/right atrial relationship, and the challenges of the dynamics and variability of TR. In addition, these devices do not treat the primary lesion, and the proof of concept remains to be seen.

## Transcatheter Tricuspid Intervention Lessons

Transcatheter tricuspid valve interventions, whether repair or replacement, may sometimes be unsuccessful in treating tricuspid valve disease. Many limitations have been described. In some cases, the tricuspid valve may not be fully corrected during the procedure, which can lead to residual leakage or dysfunction. This may have limited clinical efficacy and potential for progression despite early success. TTV interventions are associated with a risk of procedural complications such as bleeding, infection, or damage to surrounding structures. If these complications occur, they will impact and affect the relative success of the intervention. If the replacement or repair device used during the procedure is not positioned correctly or malfunctions, it can result in a failed intervention and potentially limit subsequent reintervention options. Despite the popularity of the lifetime management discussion, the best strategy will be a single durable and effective intervention. Lastly, the success of TTV interventions will depend on operator and team experience as well as the patient’s individual health status, comorbidities, and consequences of TR.^35^

The current paradigms and dogma that exist for treating TR will need to be modified, as many of these are based on experience and concepts taken from open heart surgery, left-sided heart valve interventions, and medical management for left-sided heart disease. Waiting until patients have advanced consequences of severe TR, including liver and kidney disease, chronic fatigue, and weight loss, is too late. Given that transcatheter interventions for TR are exceedingly safe, especially for repair, referral and intervention will need to be considered much earlier than the current practice. In addition, diuretic therapy is not a meaningful treatment or cure, but it does treat congestion and delays the inevitable, which is progressive TR.

If a TTV intervention fails, other treatment options may need to be considered. In some cases, additional procedures may be performed to attempt to correct the valve, or the patient may be referred for traditional open-heart surgery to repair or replace the tricuspid valve. It is important for patients to have a thorough discussion with their heart team about the risks and benefits of different treatment options and to have realistic expectations about the potential outcomes of TTV interventions.

## Conclusion

The future of transcatheter tricuspid regurgitation treatment is promising, with ongoing research and development efforts aimed at improving the safety and efficacy of these procedures. TTV interventions currently involve the use of a range of devices, including prosthetic valves and devices to repair the existing valve. Ongoing efforts are underway to improve the design and functionality of these devices, including the development of new materials and technologies to enhance durability and performance. As more experience is gained with TTV interventions, the ability to identify and select the most appropriate patients for these procedures is likely to improve. This may involve the use of more advanced diagnostic techniques and a greater understanding of the patient factors that are most predictive of treatment success. It is possible that TTV interventions may be used in combination with other therapies, such as medications or other interventional procedures, to optimize treatment outcomes.

Current transcatheter devices have been adopted from technologies designed to treat the aortic or mitral valves and may not be optimally suited for the tricuspid valve. Ongoing research into the development of dedicated tricuspid valve devices may offer improved performance and outcomes. As more clinical trials are conducted and longer-term follow-up data become available, a greater understanding of the safety and efficacy of transcatheter tricuspid valve interventions will be possible. This information is important for guiding future developments and improving patient outcomes.

Overall, the future of transcatheter tricuspid regurgitation treatment looks promising, with ongoing research and development aimed at improving patient outcomes and expanding the range of treatment options available.

## Key Points

- Studies show that the effect of tricuspid regurgitation (TR) is marginal regarding mortality but it has a significant negative impact on quality of life and healthcare burden.
- Despite overwhelming evidence for safety and effectiveness of multiple catheter-based strategies and devices to treat tricuspid valve disease, none have FDA approval as a primary strategy for tricuspid valve replacement or repair.
- Transcatheter tricuspid valve repair (TTVR) is an evolving procedure that is still being evaluated in clinical trials, and candidates for TTVR trials are typically patients with tricuspid valve disease that is severe enough to cause symptoms but who cannot undergo traditional open-heart surgery due to age, frailty, or other health conditions.
- Waiting until patients have advanced consequences of severe TR (ie, liver and kidney disease, chronic fatigue, and weight loss) is too late; given that transcatheter interventions for TR are exceedingly safe, especially for repair, referral and intervention will need to be considered much earlier than the current practice.
